# Double Bolus Alteplase Therapy during Cardiopulmonary Resuscitation for Cardiac Arrest due to Massive Pulmonary Embolism Guided by Focused Bedside Echocardiography

**DOI:** 10.1155/2018/7986087

**Published:** 2018-03-19

**Authors:** Hafiz B. Mahboob, Bruce W. Denney

**Affiliations:** ^1^University of Nevada School of Medicine, Reno, NV, USA; ^2^Renown Regional Medical Center, Reno, NV, USA

## Abstract

Massive pulmonary embolism (PE) frequently leads to cardiac arrest (CA) which carries an extremely high mortality rate. Although available, randomized trials have not shown survival benefits from thrombolytic use. Thrombolytics however have been used successfully during resuscitation in clinical practice in multiple case reports and in retrospective studies. Recent resuscitation guidelines recommend using alteplase for PE related CA; however they do not offer a standardized treatment regimen. The most consistently applied approach is an intravenous bolus of 50 mg tissue plasminogen activator (t-PA) early during cardiopulmonary resuscitation (CPR). There is no consensus on the subsequent dosing. We present a case in which two 50 mg boluses of t-PA were administered 20 minutes apart during CPR due to persistent hemodynamic compromise guided by bedside echocardiogram. The patient had an excellent outcome with normalization of cardiac function and no neurologic sequela. This case demonstrates the benefit of utilizing bedside echocardiography to guide administration of a second bolus of alteplase when there is persistent hemodynamic compromise despite achieving return of spontaneous circulation after the initial bolus, and there is evidence of persistent right ventricle dysfunction. Future trials are warranted to help establish guidelines for thrombolytic use in cardiac arrest to maximize safety and efficacy.

## 1. Introduction

Cardiac arrest due to PE is a lethal condition [1–5]. Thrombolytics have been used successfully, mostly late in CPR after initial unresponsiveness to traditional advanced cardiac life support (ACLS) [6–11]. Recent resuscitation guidelines advocate using alteplase in PE related cardiac arrest [12–17]. There are no clear guidelines or protocols for the administration of thrombolytics during CPR. We present an approach of double bolus therapy during CPR guided by focused bedside echocardiography. This case suggests the potential benefit of using bedside echocardiography to guide the administration of a second bolus of alteplase given the excellent outcome in this case.

## 2. Case Report

A 56-year-old, previously healthy Caucasian female presented to the emergency department (ED) with acute, severe shortness of breath and pleuritic chest pain for two hours with pulse of 140/min and respiratory rate of 30/min. She had undergone total right knee replacement surgery two weeks prior. She was taking aspirin 81 mg twice daily but no other medications and had no other medical history. She was brought in via ambulance in severe respiratory distress, diaphoretic, and a room air oxygen saturation of 70% and complained “I cannot breathe.” There was some bruising at the surgical site and a small right knee effusion, but the surgical site was well healed with no erythema or purulence. Her electrocardiogram (ECG) showed sinus tachycardia without any acute ischemic changes.

She was immediately given 5000 units of intravenous heparin due to the high suspicion of pulmonary embolism. Her oxygen saturations remained around 70–79% despite receiving 100% oxygen (15 L) via nonrebreather mask. Arterial blood gas (ABG) on 100% oxygen showed (pH 6.8, pCO2 58.6, and PaO2 188) severe acidosis with an elevated A-a gradient of 334.8 mmHg. Patient was emergently intubated due to persistent respiratory distress, increased work of breathing, continued air hunger, and worsening respiratory acidosis with hypercapnia despite being on 100% oxygen via nonrebreather mask. She was given etomidate and succinylcholine and intubated without difficulty.

**Figure 1:**
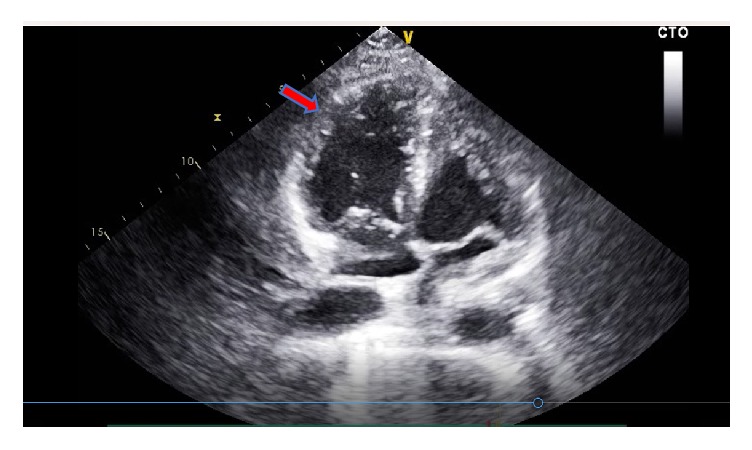
Transthoracic echocardiogram obtained before administration of t-PA: apical views showing a severely dilated right ventricle (RV) with reduced right ventricular systolic function. RV free wall is hypokinetic and RV apical wall is hypercontractile (arrow; McConnell sign). Left ventricular size and systolic function are normal.

Shortly after intubation, she underwent cardiac arrest with pulseless electrical activity (PEA). Immediate CPR was started. She received 50 mEq intravenous (IV) sodium bicarbonate, 1 mg of IV epinephrine, and 1 mg of IV atropine with four cycles of chest compression during first round of resuscitation. Emergent bedside echocardiogram was performed at the first pulse check and showed a severely dilated right ventricle (RV), with reduced right ventricular systolic function and normal left ventricular (LV) size and systolic function. These findings were suggestive of a massive PE (Figure 1 and videos 1, 2). The first bolus of alteplase 50 mg IV was given at the fifth minute into CPR with ongoing chest compression. She had return of spontaneous circulation (ROSC) one minute following the t-PA bolus. However, she went into cardiac arrest again, approximately at 15 minutes into the code with recurrent PEA. During the second round of resuscitation, she received 1 mg of epinephrine with three cycles of chest compression and had ROSC after four minutes of CPR.

However, despite achieving the ROSC, she was hypotensive and therefore norepinephrine infusion was started. Echocardiogram was still showing persistent evidence of RV dysfunction with normal LV function. Given her persistent hemodynamic compromise with recurrent cardiac arrest, the decision was made to administer a second bolus of 50 mg alteplase, which was given at 24 minutes into the code. She developed PEA for a third time approximately at 29 minutes into the code. During third round of resuscitation, she received 1 mg of epinephrine and had ROSC. She required CPR for a total of 32 minutes, with three rounds of resuscitation and 2 IV boluses of 50 mg alteplase, 20 minutes apart. Her chest X-ray at this point showed bilateral perihilar opacities with mild cardiomegaly, consistent with pulmonary edema. Her other laboratory findings are summarized in the table (Table 1).

After the third round of CPR, she also received a transfusion of two units of packed red blood cells via Belmont rapid infuser due to anemia. Bicarbonate infusion was initiated due to severe acidosis. Full dose heparin anticoagulation was started per postthrombolytics protocol. Some ecchymosis and swelling were noticed at the recent knee surgical site but she did not develop any significant bleeding complications after thrombolytic therapy. Venous Doppler ultrasound was done later which was positive for deep venous thrombosis in the gastrocnemius and popliteal veins of the right lower extremity. She was then transferred to the intensive care unit where bronchoscopy showed small pink frothy septum consistent with pulmonary edema but no evidence of aspiration.

**Figure 2:**
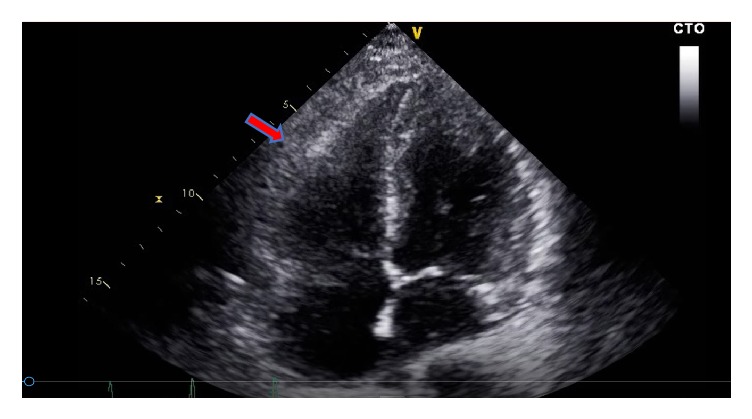
Transthoracic echocardiogram 24 hours after t-PA administration: showing improvement in RV size (arrow) and function but still dilated.

Repeat echocardiogram the next day showed improvement in RV size and function (Figure 2 and video 3). She remained on vasopressor support, bicarbonate infusion, and epoprostenol (continuous inhalation at a rate of 360 mcg/hour) due to hypotension, acidosis, and hypoxia, respectively, which were titrated off over 24 hours.

**Figure 3:**
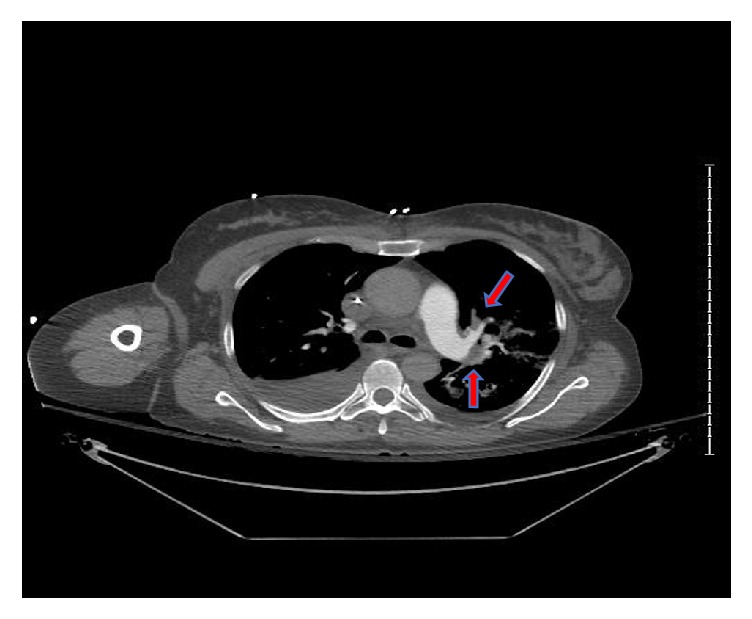
CT angiogram showing pulmonary embolism (arrow).

Subsequent computed tomography angiogram of the chest performed eighty-four hours after CA showed segmental PE in right middle and left lower lobe with scattered air space opacities, atelectasis, and pleural effusions (Figure 3).

**Figure 4:**
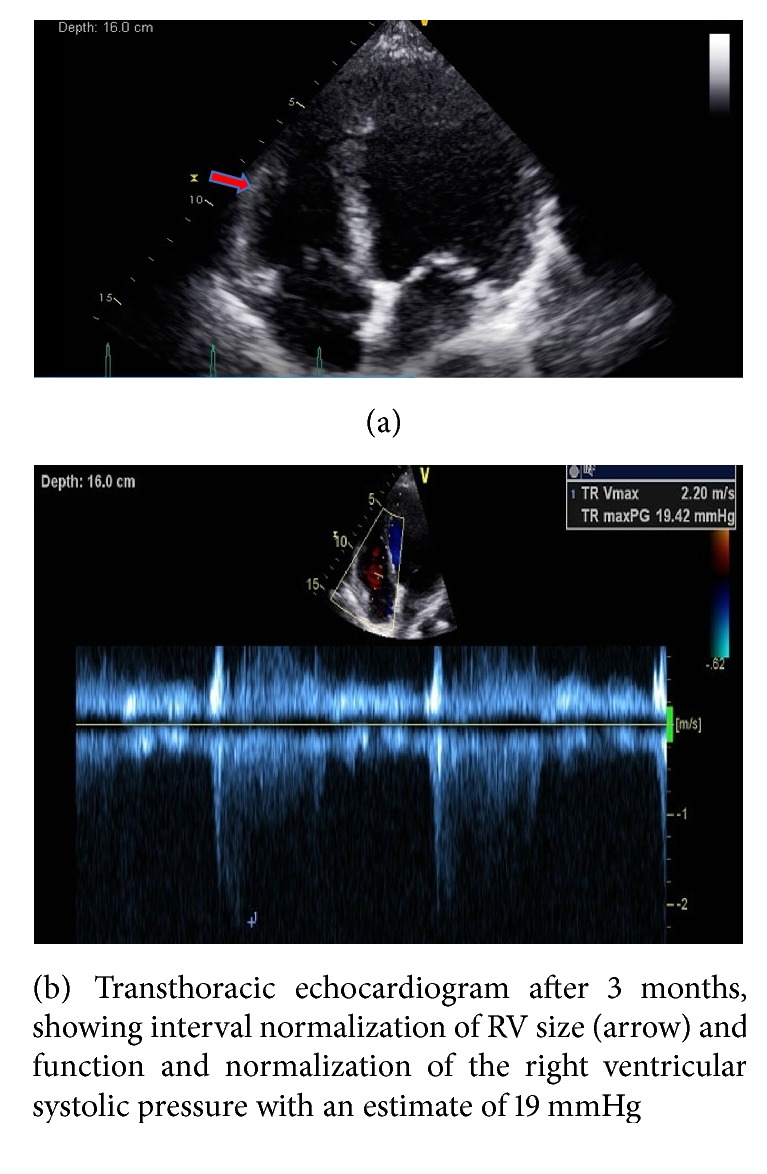


She had an excellent outcome without any neurologic sequelae or any bleeding complications. She was liberated from mechanical ventilation on hospital day 3 and supplemental oxygen was gradually weaned from high flow nasal cannula to room air by the time of discharge. Apixaban was initiated on hospital day 3. She was discharged home on hospital day 5, ambulating independently, on room air and on apixaban. On 3 months' follow-up, she had complete normalization of right ventricular size and systolic function without any residual pulmonary hypertension (Figures 4(a) and 4(b) and videos 4, 5).

## 3. Discussion

Pulmonary embolism is the third most frequent cardiovascular disease in the United States (US) and has an extremely high morbidity and mortality [1–3]. Massive pulmonary embolism can cause cardiac arrest in 41% of cases and this is the major predictor of PE related mortality, which ranges from 65% to 95% [4, 5]. Pulmonary embolism is responsible for 2% to 15% of unexpected sudden deaths [4, 5], 2% of all cardiac arrests (CA) cases, and 6.5% of CA cases of extracardiac origin [5]. Moreover, in clinical setting, pulmonary embolism is often not suspected and is underestimated as a cause of acute cardiopulmonary collapse [18, 19].

Massive PE causes sudden increase in pulmonary vascular resistance (PVR) and mean pulmonary arterial pressure (mPAP) which is proportional to degree of obstruction in patients without preexisting pulmonary vascular disease [1]. It leads to increased RV wall tension and RV failure [20]. Eventually it leads to obstructive cardiogenic shock due to decrease in LV preload [21]. Presence of RV hypokinesis in patients with acute PE is associated with significantly increased mortality and a 14% risk for having a recurrent PE [2, 22, 23]. Massive PE can cause acute myocardial infarction (AMI) [24]. Massive PE can also lead to various types of cardiac arrhythmias including PEA, asystole, and ventricular fibrillation [25–30].

Traditional ACLS and CPR have been the common practice for PE related cardiac arrest [31, 32]. Vasopressor support and anticoagulation are used as well. However, only heparinization will not affect the clot burden and hemodynamic insult acutely. Systemic thrombolytic agents can minimize clot burden by clot lysis and can decrease the risk of recurrent Pes, and therefore long term pulmonary hypertension [33]. However, majority of benefit from thrombolytic therapy in patients with pulmonary embolism is limited to hemodynamic compromise [34], and fibrinolytics are not recommended in normotensive patients [35]. Catheter guided clot lysis can be considered in case of contraindication to systemic thrombolysis. Surgical embolectomy is reserved for unstable patients who have failed maximal medical treatment or in case of contraindication to thrombolytics [36].

Unfortunately, there are no strong prospective studies to show a survival benefit for the use of fibrinolytic drugs in cardiac arrest due to massive PE. Two available randomized control trials have failed to show a statistically significant outcome [37, 38]. However, these studies had various limitations such as late administration of thrombolytics and small sample size. Overall, in clinical practice, systemic thrombolytic therapy is less frequently used for a multitude of reasons; they include (a) limited evidence, (b) risk of bleeding with high dose of thrombolytics [39], (c) especially having ongoing CPR [40], and (d) lack of specific guidelines regarding thrombolytic dosing and timing.

Thrombolytic therapy also carries 9–22% risk of major bleeding, including a 1–5% risk of intracranial hemorrhage [2, 33, 40–42]. A nonrandomized study suggested a relative contraindication to thrombolytic use for CPR duration > 10 minutes. This study analyzed use of thrombolytics in cardiac arrest due to AMI and concluded that thrombolysis could safely be applied to patients who undergo a CPR of <10 min. They did not include patients with CPR > 10 mins in their study [37]. Janata et al. in their study showed that CPR duration of >10 min did not have any impact on major bleeding complications in patients receiving thrombolytic therapy [6].

There have been numerous cases reported in American and European literature, where thrombolysis (various formulations and regimens) was used in confirmed PE or clinically suggestive history of PE related cardiac arrest cases. Thrombolytic therapy has showed very favorable outcomes even when administered late during the CPR and as slow infusions after a prolonged CPR [6–11, 24–26]. Even though the available evidence for the utility of thrombolytics in such instances is of low quality, but due to the extremely high mortality of PE related cardiac arrest and frequently reported success with thrombolytics, its use is being advocated in recent resuscitation guidelines [12–17].

Unfortunately, there is no consensus regarding the dosage and timing of thrombolytics during resuscitation. The American Heart Association (AHA) recommends a two-hour infusion of 100 mg of alteplase in those with hemodynamic compromise. However, they do not clearly address the issue of cardiac arrest [15]. The European Resuscitation Council (ERC) and European Society of Cardiology (ESC) recommend a dose of 100 mg alteplase over 2 hours or 0.6 mg/kg over 15 minutes, though again they do not specifically address the approach in cardiac arrest [12]. The British Thoracic Society in 2003 recommended a 50 mg bolus of IV alteplase, which is the regimen most frequently used in recent published literature [10, 14, 16, 17].

However, there is no existing consensus or established guidance for subsequent approach. Surgical embolectomy or catheter guided interventions can be considered for unstable patients or those who have failed maximal medical treatment [34, 36]. Fengler and Brady in their review suggested administering a second bolus of alteplase [43].

We used focused bedside echocardiogram in our patient, as she was hemodynamically unstable and was experiencing recurrent cardiac arrest. Based on our echocardiogram findings of persistent RV dysfunction and dilatation with persistent hemodynamic compromise, we administered second bolus of t-PA with excellent outcome. This emphasizes the potential implacability of echocardiography for decision-making in such situations.

Echocardiography has limited sensitivity and specificity for the diagnosis of acute PE [44]. However, transthoracic echocardiography can be helpful to identify acute PE related right ventricular dysfunction [45]. Echocardiographic findings of RV strain include dilation of RV, flattened interventricular septum (D-sign of interventricular septal shift), and the classic sign of RV apical wall hypercontractility with hypokinesis of the RV free wall and base termed as “McConnell” sign [45–47]. McConnell sign is only 77% sensitive but has a specificity of 94% for acute PE, as RV failure due to chronic pulmonary hypertension typically shows global hypokinesia [47].

Presence of RV strain has more than twofold increase in risk of early mortality compared with patients with no signs of RV strain [34]. Use of echocardiography in diagnosis and management of hemodynamically unstable patients have also been recommended by the ESC [48]. A recent study showed that the average time from beginning of CPR to get a suitable echo image was 3.9 minutes (17 seconds to 10 minutes) [49].

We used focused echocardiography to guide repeat alteplase bolus administration, as our patient remained hemodynamically unstable, despite successful ROSC after initial alteplase bolus. Fortunately, despite prolonged CPR, our patient had an excellent outcome with no cardiac or neurologic sequela. At three months' follow-up, the patient had complete normalization of right RV function without any residual pulmonary hypertension (Figures 4(a) and 4(b) and videos 4, 5).

## 4. Conclusion

Recent resuscitation guidelines recommend using thrombolytic therapy during resuscitation in cases of CA due to massive PE [1, 15–19]. Most consistently applied approach is 50 mg intravenous alteplase early during CPR. There is no existing consensus on subsequent approach. We present a case of double bolus alteplase guided by focused bedside echocardiography. Echocardiographic evidence of persistent RV dysfunction and dilatation in the setting of persistent hemodynamic instability, recurrent arrest, or even failure to achieve ROSC with initial bolus may warrant the administration of second bolus of alteplase. Future well-designed trials are needed to establish guidelines for thrombolytic therapy in CA to maximize safety and efficacy.

## Figures and Tables

**Table 1:** Patient characteristics.

Variable	Value	Target range
Pulse	140/minute	60–100/minute
Respiratory rate	34–50/minute	12–14/minute
Blood pressure	109/67 mmHg	120/80 mmHg
Temperature	97°F	97–99°F
Oxygen saturation		
Room air	70%	88–100%
100% oxygen	70–79%	88–100%
ABG on 15 L oxygen nonrebreather (NRB)		
Ph	6.8	7.4
pO2	188 mmHg	100 mmHg
pCO2	58.6 mmHg	40 mmHg
HCO3	9.2 mmol/L	25 mmHg
Laboratory data		
WBC	4.5 × 10^9^/L	4–10 × 10^9^/L
Neutrophil	73%	50–60%
Hemoglobin	7.2 g/dl	12–14 g/dl
Hematocrit	22.83%	35–45%
Platelets	133 × 10^9^/L	130–450 × 10^9^/L
Sodium	140 mEq/L	135–145 mEq/L
Potassium	4.5 mEq/L	3.6–5 mEq/L
Chloride	96 mEq/L	98–110 mEq/L
CO2	19 mEq/L	25 mEq/L
BUN	17 mg/dL	25 mg/dL
Creatinine	0.96 mg/dL	1.0 mg/dL
Anion gap	15 mEq/L	10–12 mEq/L
Lactic acid	4.4 mmol/L	<2.0 mmol/L
Troponin	4.2 ng/mL	<0.03 ng/mL
Calcium	6.6 mg/dL	8 mg/dL
Ionized calcium	<1.0 mmol/L	1.1 mmol/L
AST	372 U/L	45 U/L
ALT	214 U/L	40 U/L
ALP	156 U/L	100 U/L
T. bilirubin	1.0 mg/dL	1.0 mg/dL
CPK	203 IU/L	50 IU/L

ABG: arterial blood gas.

## Supplementary Materials

Supplementary Materials Videos  1, 2: transthoracic echocardiogram during the CPR before administration of t-PA, showing a severely dilated right ventricle (RV) with reduced systolic function. RV free wall is hypokinetic and apical wall is hypercontractile (McConnell sign). There is flattening of interventricular septum with paradoxical motion of septum in diastole towards left ventricle (D-shaped left ventricle) consistent with significant RV overload. There is a dilated right atrium and moderate tricuspid regurgitation. Left ventricular size and systolic function are normal. Video  3: transthoracic echocardiogram 24 hours after t-PA administration, showing improvement in RV size and function but still dilated. Videos  4-5: transthoracic echocardiogram after 3 months showing interval normalization of right ventricular size and function. Right ventricular systolic pressure has also normalized.

## References

[1] Gerges C., Skoro-Sajer N., Lang I. M. (2014). Right ventricle in acute and chronic pulmonary embolism (2013 grover conference series). *Pulmonary Circulation*.

[2] Goldhaber S. Z., Visani L., de Rosa M. (1999). Acute pulmonary embolism: clinical outcomes in the International Cooperative Pulmonary Embolism Registry (ICOPER). *The Lancet*.

[3] Bergamo C. (2014). Thrombolysis for pulmonary embolism and risk of all-cause mortality, major bleeding, and intracranial hemorrhage: A meta-analysis: Chatterjee S, Chakraborty A, Weinberg A, et al. JAMA 2014;311(23):2414-21.. *The Journal of Emergency Medicine*.

[4] Gallerani M., Manfredini R., Ricci L. (1992). Sudden death from pulmonary thromboembolism: Chronobiological aspects. *European Heart Journal*.

[5] Kuisma M., Alaspaa A. (1997). Out-of-hospital cardiac arrests of non-cardiac origin. Epidemiology and outcome. *European Heart Journal*.

[6] Janata K., Holzer M., Kürkciyan I. (2003). Major bleeding complications in cardiopulmonary resuscitation: the place of thrombolytic therapy in cardiac arrest due to massive pulmonary embolism. *Resuscitation*.

[7] Zhu T., Pan K., Wang Y. (2015). Successful resuscitation with thrombolysis of pulmonary embolism due to thrombotic thrombocytopenic purpura during cardiac arrest. *The American Journal of Emergency Medicine*.

[8] Hsin T., Chun F. W., Tao H. L. (2014). Ultra-long cardiopulmonary resuscitation with thrombolytic therapy for a sudden cardiac arrest patient with pulmonary embolism. *The American Journal of Emergency Medicine*.

[9] Gupta R., Jindal A., Cranston-D′Amato H. (2014). Benefits of thrombolytics in prolonged cardiac arrest and hypothermia over its bleeding risk. *International Journal of Critical Illness & Injury Science*.

[10] Yin Q., Li X., Li C. (2015). Thrombolysis after initially unsuccessful cardiopulmonary resuscitation in presumed pulmonary embolism. *The American Journal of Emergency Medicine*.

[11] Sharifi M., Vajo Z., Javadpoor S. (2016). Pulseless electrical activity in pulmonary embolism treated with thrombolysis (from the “PEAPETT” study). *The American Journal of Emergency Medicine*.

[12] Konstantinides S. V., Torbicki A., Agnelli G. (2014). 2014 ESC Guidelines on the diagnosis and management of acute pulmonary embolism: The Task Force for the Diagnosis and Management of Acute Pulmonary Embolism of the European Society of Cardiology (ESC)Endorsed by the European Respiratory Society (ERS). *European Heart Journal*.

[13] Soar J., Perkins G. D., Abbas G. (2010). European Resuscitation Council guidelines for resuscitation 2010 section 8. Cardiac arrest in special circumstances: electrolyte abnormalities, poisoning, drowning, accidental hypothermia, hyperthermia, asthma, anaphylaxis, cardiac surgery, trauma, pregnancy, electrocution. *Resuscitation*.

[14] Lavonas E. J., Drennan I. R., Gabrielli A. (2015). Part 10: special circumstances of resuscitation: 2015 American Heart Association guidelines update for cardiopulmonary resuscitation and emergency cardiovascular care. *Circulation*.

[15] Jaff M. R., McMurtry M. S., Archer S. L. (2011). Management of massive and submassive pulmonary embolism, iliofemoral deep vein thrombosis, and chronic thromboembolic pulmonary hypertension: a scientific statement from the american heart association. *Circulation*.

[16] British Thoracic Society Standards of Care Committee Pulmonary Embolism Guideline Development Group (2003). British Thoracic Society guidelines for the management of suspected acute pulmonary embolism. *Thorax*.

[17] O’Connor G., Fitzpatrick G., El-Gammal A., Gilligan P. (2015). Double Bolus Thrombolysis for Suspected Massive Pulmonary Embolism during Cardiac Arrest. *Case Reports in Emergency Medicine*.

[18] Stein P. D. (2000). Silent pulmonary embolism. *JAMA Internal Medicine*.

[19] Bougouin W., Marijon E., Planquette B. (2016). Factors Associated with Pulmonary Embolism-Related Sudden Cardiac Arrest. *Circulation*.

[20] Pruszczyk P., Bochowicz A., Torbicki A. (2003). Cardiac troponin T monitoring identifies high-risk group of normotensive patients with acute pulmonary embolism. *CHEST*.

[21] Lualdi J. C., Goldhaber S. Z. (1995). Right ventricular dysfunction after acute pulmonary embolism: Pathophysiologic factors, detection, and therapeutic implications. *American Heart Journal*.

[22] Alpert J. S., Smith R., Carlson C. J., Ockene I. S., Dexter L., Dalen J. E. (1976). Mortality in Patients Treated for Pulmonary Embolism. *Journal of the American Medical Association*.

[23] Miller R. L., Das S., Anandarangam T. (1998). Association between right ventricular function and perfusion abnormalities in hemodynamically stable patients with acute pulmonary embolism. *CHEST*.

[24] Toprak C., Avci A., Ozturkeri B., Tabakci M. M., Kahveci G. (2014). PE with ST-segment elevation in leads V1 -3 and AVR treated successfully by catheter directed high-dose bolus thrombolytic therapy during CPR. *The American Journal of Emergency Medicine*.

[25] Marzegalli M., Rietti P., Chirico M. A. (1994). Heart arrest in acute pulmonary embolism: An anatomo-clinical study. *Giornale Italiano Di Cardiologia*.

[26] Kürkciyan I., Meron G., Sterz F. (2000). Pulmonary embolism as a cause of cardiac arrest: presentation and outcome. *JAMA Internal Medicine*.

[27] Deasy C., Bray J. E., Smith K., Harriss L. R., Bernard S. A., Cameron P. (2011). Out-of-hospital cardiac arrests in young adults in Melbourne, Australia-Adding coronial data to a cardiac arrest registry. *Resuscitation*.

[28] Akinboboye O. C., Brown E. J., Queirroz R. (1993). Recurrent pulmonary embolism with second-degree atrioventricular block and near syncope. *American Heart Journal*.

[29] Koutkia P., Wachtel T. J. (1999). Pulmonary embolism presenting as syncope: Case report and review of the literature. *Heart & Lung: The Journal of Acute and Critical Care*.

[30] Hess E. P., Campbell R. L., White R. D. (2007). Epidemiology, trends, and outcome of out-of-hospital cardiac arrest of non-cardiac origin. *Resuscitation*.

[31] Stein P. D., Matta F. (2012). Thrombolytic therapy in unstable patients with acute pulmonary embolism: Saves lives but underused. *American Journal of Medicine*.

[32] Lin B. W., Schreiber D. H., Liu G. (2012). Therapy and outcomes in massive pulmonary embolism from the Emergency Medicine Pulmonary Embolism in the Real World Registry. *The American Journal of Emergency Medicine*.

[33] Arcasoy S. M., Kreit J. W. (1999). Thrombolytic therapy of pulmonary embolism: A comprehensive review of current evidence. *CHEST*.

[34] Ten Wolde M., Söhne M., Quak E., Mac Gillavry M. R., Büller H. R. (2004). Prognostic value of echocardiographically assessed right ventricular dysfunction in patients with pulmonary embolism. *JAMA Internal Medicine*.

[35] Konstantinides S., Geibel A., Olschewski M. (1997). Association between thrombolytic treatment and the prognosis of hemodynamically stable patients with major pulmonary embolism: Results of a multicenter registry. *Circulation*.

[36] Schmid C., Zietlow S., Wagner T. O. F., Laas J., Borst H. G. (1991). Fulminant pulmonary embolism: Symptoms, diagnostics, operative technique, and results. *The Annals of Thoracic Surgery*.

[37] Böttige B. W., Böhrer H., Bach A., Motsch J., Martin E. (1994). Bolus injection of thrombolytic agents during cardiopulmonary resuscitation for massive pulmonary embolism. *Resuscitation*.

[38] Abu-Laban R. B., Christenson J., Innes G. D. (2002). Tissue plasminogen activator in cardiac arrest with pulseless electrical activity. *The New England Journal of Medicine*.

[39] Klinge U., Klosterhalfen B., Tons C. (1991). A bleeding complication as a consequence of bolus lysis after resuscitation. *Deutsche Medizinische Wochenschrift*.

[40] Tenaglia A. N., Califf R. M., Candela R. J. (1991). Thrombolytic therapy in patients requiring cardiopulmonary resuscitation. *American Journal of Cardiology*.

[41] Diehl J.-L., Meyer G., Igual J. (1992). Effectiveness and safety of bolus administration of alteplase in massive pulmonary embolism. *American Journal of Cardiology*.

[42] Hamel E., Pacouret G., Vincentelli D. (2001). Thrombolysis or heparin therapy in massive pulmonary embolism with right ventricular dilation: Results from a 128-patient monocenter registry. *CHEST*.

[43] Fengler B. T., Brady W. J. (2009). Fibrinolytic therapy in pulmonary embolism: an evidence-based treatment algorithm. *The American Journal of Emergency Medicine*.

[44] Miniati M., Monti S., Pratali L. (2001). Value of transthoracic echocardiography in the diagnosis of pulmonary embolism: results of a prospective study in unselected patients. *American Journal of Medicine*.

[45] MacCarthy P., Worrall A., McCarthy G., Davies J. (2002). The use of transthoracic echocardiography to guide thrombolytic therapy during cardiac arrest due to massive pulmonary embolism. *Emergency Medicine Journal*.

[46] Lodato J. A., Ward R. P., Lang R. M. (2008). Echocardiographic predictors of pulmonary embolism in patients referred for helical CT. *Journal of Echocardiography*.

[47] McConnell M. V., Solomon S. D., Rayan M. E., Come P. C., Goldhaber S. Z., Lee R. T. (1996). Regional right ventricular dysfunction detected by echocardiography in acute pulmonary embolism. *American Journal of Cardiology*.

[48] Konstantinides S. V., Torbicki A., Agnelli G. (2015). Erratum: 2014 ESC Guidelines on the diagnosis and management of acute pulmonary embolism (European Heart Journal (2014) 35 (3033-73) DOI 10.1093/eurheartj/ehu283. *European Heart Journal*.

[49] Varriale P., Maldonado J. M. (1997). Echocardiographic observations during inhospital cardiopulmonary resuscitation. *Critical Care Medicine*.

