# Supplementary material for: Double Bolus Alteplase Therapy during Cardiopulmonary Resuscitation for Cardiac Arrest due to Massive Pulmonary Embolism Guided by Focused Bedside Echocardiography
Source: Case Rep Crit Care. 2018 Mar 19;2018:7986087. doi: 10.1155/2018/7986087 (PMC5884296; doi:10.1155/2018/7986087)
Supplement: Supplementary Materials — Videos 1, 2: transthoracic echocardiogram during the CPR before administration of t-PA, showing a severely dilated right ventricle (RV) with reduced systolic function. RV free wall is hypokinetic and apical wall is hypercontractile (McConnell sign). There is flattening of interventricular septum with paradoxical motion of septum in diastole towards left ventricle (D-shaped left ventricle) consistent with significant RV overload. There is a dilated right atrium and moderate tricuspid regurgitation. Left ventricular size and systolic function are normal. Video 3: transthoracic echocardiogram 24 hours after t-PA administration, showing improvement in RV size and function but still dilated. Videos 4-5: transthoracic echocardiogram after 3 months showing interval normalization of right ventricular size and function. Right ventricular systolic pressure has also normalized. [file 7986087.f1.docx]

Supplementary Materials:

Echocardiogram videos:

Video: 1 to 5
